# A Victim of Cocaine

**Published:** 1889-07

**Authors:** 


A VICTIM OF COCAINE.—"Give me the sweet cocaine." These were the last words of Dr. J. W. Underhill a minute before he died a few days ago. Miles Beatty, his faithful attendant, handed him the bottle. Trembling like an aspen leaf he convulsively grasped it, but could not get the bottle to his lips. His teeth were clenched together. He hung on to his attendant as he leaned back against several pillows. The attendant put the bottle to his lips. The dying man made a desperate struggle to force his tongue to the fascinating liquid, but the angel of death stood over his shoulder, and the teeth were as firmly glued together as the sutures of a skull and the mouth would not open. A second of exhaustion now came over the dying man, then a spasm, and, grasping his attendant by the neck, his heart stopped, his pulse ceased, he fell back limp and was dead.

Like all habitual cocaine slaves, the doctor was subject to horrible delusions. He imagined that somebody, some enemy, was continually pursuing him, trying to kidnap him. Delirium tremens patients frequently imagine they see horrible animals, gigantic monsters all mouth and no body, of all colors, and with numerous legs and claws, but the doctor had none of these. His hallucination was always the same. Some enemy was after him.

The death certificate will read "death from lockjaw," as that was the immediate cause of his death. A week ago Monday, while eating with a rusty silver-plated fork, he dropped it with sufficient force to penetrate the exterior portion of the left ankle. Only one prong penetrated the flesh. No blood came from the wound, which healed. Yet afterward the limb became swollen, and symptoms of blood poisoning set in.

A specialist in cocaine last year predicted that his death would be a sudden one, probably from pneumonia or pleurisy, or an accident, which a person with a less impaired constitution would resist and throw off. And so it was. The slight wound from the fork, which a man in perfect health would have paid no attention to, in the doctor's case, with his constitution sapped by the use of cocaine, proved fatal.

The quantity of cocaine he could take in a day amounted to forty or fifty grains. He wanted it every half-hour or hour, then would sleep for half an hour or an hour. He generally took it in three or five grain doses. It produced a sort of stupor, and it was no unc mmon thing for the children of the house to enter his room and find him in a doze, conscious, yet stupefied.

He told a curious tale about his becoming addicted to the habit. He was at one time a director of the Cincinnati Hospital, and about the time the anæsthetic quality of cocaine was discovered (in 1884) some leading physicians, he said, wished to try the experiment on him. He was hale and hearty, had no constitutional disease, no hereditary taint, and was possessed of great firmness of character and will power. He tried it, and found it to be an octupus out of whose grasp by no power of his own could he escape.

